# Combined spinal cord sclerosis, alcohol and bipolar disorder

**DOI:** 10.1192/j.eurpsy.2025.1254

**Published:** 2025-08-26

**Authors:** Z. Souhlal

**Affiliations:** 1 New hospital of Navarre, france, France

## Abstract

**Introduction:**

The alcohol use disorder has consequences on the metabolic, gastroenterological and especially neurological levels.

The somatic complications of alcohol use disorder add to the psychological and psychiatric complications and repercussions.

This involves presenting the case of Mr D;D admitted as part of the emergency for psychiatric evaluation.

**Objectives:**

Interest in highlighting multidisciplinary care for somatic and psychiatric liaison

**Methods:**

Presentation of clinical case

**Results:**

Patient case

**Conclusions:**

The management of patients with a psychiatric disorder or a request for psychiatric evaluation within the framework of the emergency liaison requires multidisciplinary and coordinated care.

Close collaboration allows early diagnosis and better overall patient care.

**Disclosure of Interest:**

None Declared

